# Long term changes in thrombocytopenia and leucopenia after HCV eradication with direct-acting antivirals

**DOI:** 10.1186/s12876-023-02829-w

**Published:** 2023-05-25

**Authors:** Kazuto Tajiri, Kazuhiko Okada, Hiroyuki Ito, Kengo Kawai, Yoshiro Kashii, Yoshiharu Tokimitsu, Nozomu Muraishi, Aiko Murayama, Yuka Hayashi, Masami Minemura, Terumi Takahara, Yukihiro Shimizu, Ichiro Yasuda

**Affiliations:** 1 Third Department of Internal Medicine, Faculty of Medicine, University of Toyama, Toyama, 2630 Sugitani 930-0194 Japan; 2 Gastroenterology, Toyama Red Cross Hospital, Toyama, 930-8562 Japan; 3 Gastroenterology, Takaoka Municipal Hospital, Takaoka, 933-8550 Japan; 4 Gastroenterology, Nanto Municipal Hospital, Nanto, 932-0211 Japan; 5 Gastroenterology, Saiseikai Toyama Hospital, Toyama, 931-8533 Japan

**Keywords:** Hepatitis C virus, Thrombocytopenia, Hypersplenism, Direct-acting antiviral

## Abstract

**Background:**

Thrombocytopenia due to hypersplenism is a major complication of hepatitis C virus (HCV)-associated cirrhosis. HCV eradication improves these complications in some patients, but the long-term effects of HCV eradication on these complications remain unclear, especially in patients treated with direct acting antivirals (DAAs). The aim was to evaluate long term changes in thrombocytopenia and leucopenia after HCV eradication with DAAs.

**Methods:**

The present multicenter study retrospectively evaluated changes over 5 years in thrombocytopenia and leukocytopenia, as well as changes in liver fibrosis markers and spleen size, in 115 patients with HCV-cirrhosis treated with DAAs.

**Results:**

Thrombocytopenia and leukocytopenia were improved 4 weeks after DAA administration, with thrombocytopenia show further gradual improvement over the next year. Fib-4 index was markedly reduced 1 year after DAA, followed by subsequent gradual reduction over the next 4 years. Spleen size showed gradual annual reductions, with patients experiencing spleen size reduction characterized at baseline by bilirubinemia.

**Conclusions:**

Rapid DAA-associated HCV eradication might lead to rapid disappearance of liver inflammation and bone marrow suppression due to HCV infection. HCV eradication may gradually improve portal hypertension, reducing spleen size.

**Supplementary Information:**

The online version contains supplementary material available at 10.1186/s12876-023-02829-w.

## Background

Hepatitis C virus (HCV) infection is estimated to affect 170 million people worldwide, with about one-fourth of these patients developing liver cirrhosis through chronic infection [1]. Thrombocytopenia, which is frequently observed in HCV-infected patients [2, 3], is thought to be caused by complex mechanisms, which can include increased destruction of platelets and their decreased production in bone marrow [4]. Platelet destruction is increased by hypersplenism, which is caused by HCV infection-associated portal hypertension and autoimmunity [4]. Hypersplenism is regarded as the main factor contributing to thrombocytopenia, the frequency of which increases as liver fibrosis progresses or cirrhosis develops [2, 5]. In addition, thrombocytopenia is associated with an increased risk of bleeding occurring during invasive procedures [3].

HCV eradication with interferon (IFN) or direct-acting antivirals (DAA) has been shown to improve liver fibrosis and extrahepatic manifestations of HCV infection [6, 7]. IFN eradication of HCV was found to increase platelet counts, which persisted for several years after HCV eradication [8]. DAA eradication of HCV was also found to improve thrombocytopenia, beginning 4 weeks after DAA initiation [9, 10]. IFN itself, however, can induce thrombocytopenia and have various immune-modulatory effects [11], suggesting that the difference in effects of IFN and DAA on thrombocytopenia improvement may be caused by the pharmacological effects of IFN. DAA treatment can reduce HCV-RNA viral loads immediately and eradicate infection, generally within 4 weeks, with little effect on bone marrow and host immunity [12, 13]. Thus the effects of DAA on thrombocytopenia may directly reflect the pathogenesis of HCV-induced thrombocytopenia.

Although the short-term, 12-week effects of DAA treatment on thrombocytopenia following HCV eradication have been assessed [9, 10, 14], the long term effects of DAA treatment on thrombocytopenia and its association with hypersplenism remain uncertain. The present study evaluated changes in platelet and leukocyte counts and spleen size in DAA-treated, cirrhotic HCV-infected patients for 5 years.

## Methods

### Patients

This multi-center, retrospective, observational study included patients with HCV-cirrhosis who were treated with DAA at Toyama University Hospital, Toyama Red-Cross Hospital, Takaoka Municipal Hospital, Nanto Municipal Hospital and Saiseikai Toyama Hospital between January 2014 and July 2020. Patients who did not undergo imaging evaluation, such as ultrasonography (US) or computed tomography (CT), before and after DAA treatment and those observed for less than 1-year after treatment were excluded. Patients diagnosed with active HCC and co-infection with hepatitis B virus were also excluded (supplementary Fig. 1). The baseline characteristics of patients were evaluated, including sex, age, etiology of cirrhosis, and liver function markers. Liver cirrhosis was diagnosed by hepatologists with over 20 years of experience, based on imaging modalities, such as US, CT and/or elastography, and the titers of fibrosis markers, such as platelet counts, Fib-4 index, and other fibrosis markers. Child–Pugh score, an index based on serum concentrations of albumin and bilirubin, prothrombin time, and the degree of hepatic coma and ascites, [15] and Fib-4 index, an indicator of liver fibrosis [16] were evaluated before DAA treatment. Informed consent was obtained from each patient. This multicenter study was performed in accordance with the 1975 Declaration of Helsinki and was approved by the Ethics Committee of Toyama University (approval number: R2019-131).

### Measurement of spleen size

Spleen size was determined by measuring the maximum long (a) and short (b) diameters of the spleen on US and calculating splenic index (SI), defined as a x b. The error of spleen size in SI measurement with US was evaluated in 26 patients. Furthermore, the association between spleen area and volume was also evaluated. Spleen volume assessment with US was performed according to the method reported previously (length x width x thickness × 0.523) [17]. Spleen size in patients who did not undergo US examination were evaluated by CT, with SI calculated as the CT-measured long diameter x short diameter of the spleen. The correlation between SI evaluated by US (SI-US) and by CT (SI-CT) was confirmed in patients who underwent CT examination within 6 months prior to DAA administration.

### Treatment with direct-acting antivirals and patients’ follow-up

Treatment regimens were determined by each hepatologist according to Japanese HCV treatment guidelines [18]. Treatment regimens included daclatasvir plus asunaprevir in patients with HCV genotype 1b from 2014 to 2016; sofosbuvir (SOF) plus ledipasvir in patients with HCV genotypes 1b and 2a/2b from 2015 to 2020; SOF plus ribavirin in patients with HCV genotypes 2a/2b from 2015 to 2017; and glecaprevir plus pibrentasvir in patients with any HCV genotype from 2017 to 2021. Other regimens included ombitasvir, paritaprevir and ritonavir from 2016 to 2017, elbasvir plus grazoprevir in 2017 and SOF plus velpatasvir from 2019 to 2020, depending on patient condition and the timing of treatment. A sustained viral response (SVR) was defined as complete clearance of HCV-RNA 12 weeks after the end of DAA treatment. Patients were monitored every 4 weeks during DAA treatment, and every 12 weeks thereafter. All patients underwent imaging evaluation every 24 weeks. Patients were monitored for a median 53.3 months (range: 12.1 to 74.3 months) after the end of DAA therapy.

### Statistical analyses

Variables were reported as mean ± standard deviation (SD). Categorical variables were compared by Fisher’s exact test. Continuous variables were compared by Student’s t-test or the Mann–Whitney U-test, as appropriate. Correlations between two variables were evaluated with Pearson’s correlation test. Multivariate analyses were performed with a logistic regression model. All statistical analyses were performed using SPSS software, version 19.0 (SPSS Inc., Chicago, IL, USA), with *p* < 0.05 defined as statistically significant.

## Results

### Patients characteristics

The present study included a total of 115 patients, 52 (45.2%) men and 63 (54.8%) women, of mean age 71 years (Table 1). Ninety-six (83.5%) patients were infected with HCV genotype 1b, and 29 (25.2%) had a previous history of HCC. Esophageal varices were found absent in 71 patients (61.7%) and present in 19 patients (16.5%); however, esophageal varices were not evaluated before DAA therapy in 25 patients. Leukocytopenia (mean white blood cell (WBC) count, 3.855/µL) and thrombocytopenia (mean platelet count, 10.2 × 10^4^/µL) were also observed. Patients had a mean Fib-4 index of 7.27 and a mean SI of 38.2 before DAA administration. Difference of SI measurement with US was limited in each procedure (1^st^ procedure versus 2^nd^ procedure, *n* = 26: r = 0.990, *p* < 0.001, Supplementary Fig. 2A). SI with spleen are was well correlated with SI with spleen volume (SI-volume versus SI-area, *n* = 20: r = 0.820, *p* < 0.001, Supplementary Fig. 2B). Evaluation of 59 patients before DAA treatment showed that SI-US (35.95 cm^2^) and SI-CT (36.02 cm^2^) were well correlated (r = 0.988, *p* < 0.001, Supplementary Fig. 3).

**Table 1:** Baseline patients characteristics

	Number of case, median (range)
Age (years)	71 (48–86)
Gender (M/F)	52/63
Genotype (1b/2a,2b)	96/19
History of HCC (y/n)	29/86
Esophageal varices (n/y/unknown)	71/19/25
White blood cells (/µL)	3,850 (1,460–8,920)
Platelets (× 10^4^/µL)	10.2 (3.2–24.1)
γ-GTP (U/L)	35 (13–388)
ALT (U/L)	46 (9–248)
Albumin (g/dL)	3.6 (1.6–4.3)
Total-bilirubin (mg/dL)	0.77 (0.2–1.9)
Creatinine (mg/dL)	0.70 (0.37–9.93)
Child–Pugh score	5 (5–8)
Fib-4 index	6.67 (1.85–25.39)
Splenic index (cm^2^)	36.5 (0*-80.0)

*M* Male, *F* Female, *n* No, *y* Yes, *HCC* Hepatocellular carcinoma, *γ-GTP* γ -glutamyl transpeptidase, *ALT* Alanine amino transferase

*splenectomy state

### Results of DAA treatment

DAAs were administered in accordance with treatment guidelines, with 110 (95.7%) of the 115 patients achieving SVR (Supplementary Table 1). Two patients could not complete 12 weeks of DAA treatment due to adverse events, with one patient being treated for 3 weeks and the other for 4 weeks, but both achieved SVR. Adverse events during DAA treatment including transient transaminase increases in five patients, malaise in two patients and skin eruption in one patient. Five patients who did not initially achieve SVR were switched to another DAA, with all five achieving SVR on the second regimen.

### Hematological parameters after HCV eradication with DAA

**Fig. 1:**
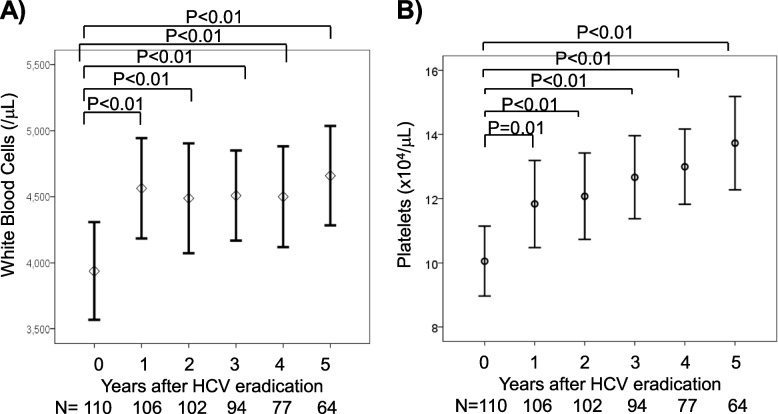
Effects of DAA treatment on **A** white blood cell counts and **B** platelet counts after HCV eradication in patients with chronic HCV infection. Five patients without HCV eradication were excluded from the analysis. Statistical difference was evaluated by paired Student’s t-test. The numbers under the graphs indicate the numbers of patients. Numbers of patients were varied due to the retrospective nature of present study. In WBC and platelets assessment, 110 patients who achieved SVR were included at 0-year (before DAA treatment), whereas 64 patients were included at 5-year after DAA treatment

Chronological changes in hematological parameters were assessed in the 110 patients who achieved SVR. One year after HCV eradication, mean WBC count, which was 3,855/µL before DAA treatment, increased to 4,546/µL (*p* < 0.01), with the increase maintained for 5 years (Fig. 1A). Platelet count, which was 10.2 × 10^4^/µL before DAA treatment, increased to 12.0 × 10^4^/µL 1-year later (*p* = 0.01), with further gradual increases to 13.8 × 10^4^/µL at 5-years (Fig. 1B). A comparison of platelet counts after DAA treatment in patients with pre-treatment platelet counts ≤ 10 × 10^4^/µL and > 10 × 10^4^/µL or not showed that platelet recovery was more prominent and maintained in patients with severe thrombocytopenia (Supplementary Fig. 4A, B).

Evaluation of hematological parameters within 24 weeks after DAA treatment showed that, although they were obtained from 63 patients in our department only, mean WBC count, which was 4,156/µL before DAA treatment, increased to 4,520/ µL 4 weeks after DAA treatment (*p* < 0.01) and was maintained for the following 20 weeks (Supplementary Fig. 5A). Moreover, mean platelet count, which was 10.2 × 10^4^/ µL before DAA treatment, increased to 11.3 × 10^4^/ µL 4 weeks after DAA treatment (*p* < 0.01) and was maintained for the following 20 weeks (Supplementary Fig. 5B).

### Changes of liver fibrosis and splenomegaly after DAA treatment

**Fig. 2:**
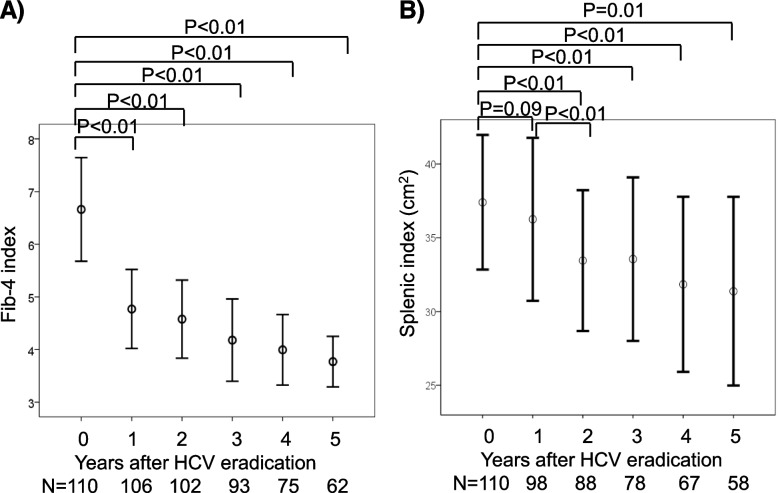
Effects of DAA treatment on **A** Fib-4 index and **B **splenic index after HCV eradication in patients with chronic HCV infection. Five patients without HCV eradication were excluded from the analysis. Statistical difference was evaluated by paired Student’s t-test. The numbers under the graphs indicate the numbers of patients. Numbers of patients were varied due to the retrospective nature of present study. In Fib-4 index and SI assessment, 110 patients who achieved SVR were included at 0-year (before DAA treatment), whereas 62 and 58 patients were included at 5-year after DAA treatment, respectively

Mean Fib-4 index, which was 6.67 before DAA treatment, was markedly lower, at 4.85, 1-year after DAA treatment (*p* < 0.01), with this index showing a further gradual decrease for up to 5 years (Fig. 2A). Similarly, mean SI, which was 37.4 before DAA treatment, was significantly decreased, to 33.1, 2-years after DAA treatment (*p* < 0.01) and gradually decreased during 5 years (Fig. 2B). Evaluation of Fib-4 index within 24 weeks after DAA treatment in our department showed that the mean index, which was 7.18 before DAA treatment, was significantly reduced to 4.84 4 weeks after DAA treatment (*p* < 0.01) and was generally maintained for the following 20 weeks (Supplementary Fig. 6).

### The significance of splenomegaly and its clinical impact

**Fig. 3:**
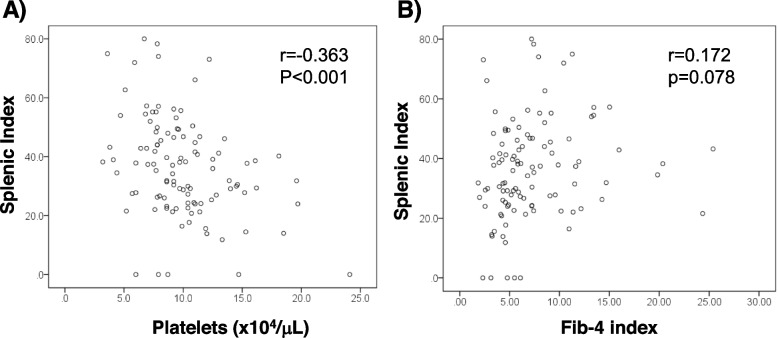
Correlations of splenic index with **A** platelet counts and **B** Fib-4 index in DAA-treated patients infected with HCV. The numbers in the right upper columns represent the correlation coefficient (r) and statistical significance (P)

To evaluate the significance of splenomegaly, correlations between SI and liver fibrosis markers were analyzed. Pretreatment SI was negatively correlated with pretreatment platelet count (r = -0.363, *p* < 0.001, Fig. 3A), but not with pretreatment Fib-4 index (r = 0.172, *p* = 0.078, Fig. 3B). A cox regression analysis to evaluate factors to be associated with SI decrease at 2-year after DAA (defined as more than 5% decrease in SI) showed that increased bilirubin is an independent contributor for SI decrease at 2-year after DAA. Higher ALT and lower Fib-4 index were weakly associated with SI-decrease (Table 2). A > 20% increase in platelet count 1-year after DAA was associated with higher ALT (Table 3). A comparison of the characteristics of patients who did and did not improve esophageal varices after DAA (F-factor decreased in endoscopic finding), it was more frequent in patients with lower SI 4 years after DAA (Supplementary Table 2).

**Table 2:** Factors for SI decrease 2-year after DAA

Variable	Odds ratio	95% CI	*p*
Age (years)	0.991	0.899–1.091	0.848
ALT (U/L)	1.023	1.000–1.046	0.053
µ-GTP (U/L)	0.997	0.985–1.009	0.578
Albumin (g/dL)	0.690	0.138–3.451	0.652
Total-bilirubin (mg/dL)	10.013	1.592–62.959	0.014
Creatinine (mg/dL)	1.432	0.857–2.393	0.170
White blood cells (/µL)	1.000	0.999–1.000	0.381
Platelets (× 10^4^/µL)	0.826	0.635–1.074	0.153
AFP (ng/mL)	1.005	0.988–1.023	0.564
Fib-4 index	0.772	0.582–1.024	0.073
Splenic Index (cm^2^)	0.986	0.938–1.037	0.577

*SI* Splenic index, *CI* Confidence interval, *DAA* Direct acting antiviral, *AL* Alanine amino transferase, *γ-GTP* γ-glutamyl transpeptidase, *AFP* Alpha fetoprotein

**Table 3:** Factors for 20% increase of platelet 1-year after DAA

Variable	Odds ratio	95% CI	*p*
Age (years)	1.068	0.949–1.201	0.273
ALT (U/L)	1.025	1.002–1.048	0.030
γ-GTP (U/L)	1.003	0.988–1.018	0.734
Albumin (g/dL)	5.609	0.790–39.830	0.085
Total-bilirubin (mg/dL)	0.827	0.142–4.804	0.833
Creatinine (mg/dL)	0.183	0.007–5.077	0.316
White blood cells (/µL)	1.000	0.999–1.000	0.753
Platelets (× 10^4^/ µL)	0.861	0.583–1.273	0.453
AFP (ng/mL)	1.013	0.996–1.031	0.141
Fib-4 index	1.022	0.734–1.423	0.898
Splenic Index (cm^2^)	1.008	0.950–1.070	0.789

*DAA* Direct acting antiviral, *CI* Confidence interval, *ALT* Alanine amino transferase, *γ-GTP* γ-glutamyl transpeptidase, *AFP* Alpha fetoprotein

## Discussion

The present study showed that DAA treatment of patients rapidly improved leukocytopenia and thrombocytopenia, with a 20% increase within 4 weeks, in patients with HCV-associated cirrhosis, followed by a gradual reduction in splenomegaly. The rapid increase in platelet count was followed by gradual yearly increases, with platelet counts being nearly 40% higher 5 years after DAA treatment than at baseline. DAA treatment resulted in yearly improvements in liver fibrosis, accompanied by improvements in thrombocytopenia and hypersplenism. Platelet transfusions have limited effects in patients with HCV cirrhosis and thrombocytopenia, with platelet counts increasing only 20% after transfusion [19]. Cirrhotic patients with thrombocytopenia have been treated with thrombopoietin (TPO) receptor agonists such as lusutrombopag a few weeks before invasive procedures [19, 20], markedly reducing the need for platelet transfusion. Although several weeks are required for DAA to increase platelet counts, the effects of HCV eradication by DAA are comparable to those of platelet transfusion.

DAA treatment has been shown to rapidly improve thrombocytopenia, with effects observed within 4 weeks [9, 10]. TPO concentration before DAA treatment was found to be associated with increased platelet counts after DAA treatment [14]. However, DAA treatment did not alter TPO concentrations within 48 weeks [10], suggesting that TPO is not a main contributor to thrombocytopenia in patients with HCV cirrhosis. The present study also showed that DAA treatment improved leukocytopenia within 4 weeks, with white blood cell counts maintained for 5 years. These findings suggest that leukocytopenia in HCV-cirrhosis is mostly caused by HCV-induced myelosuppression. HCV-RNA has been detected in bone marrow of patients with chronic HCV infection, with viral RNA inducing bone marrow suppression [21, 22]. The rapid increase in hematological parameters after DAA treatment may therefore be caused by a reduction in HCV-induced myelosuppression. Further studies are needed to determine the precise mechanism by which DAA treatment suppresses leukocytopenia.

The present study also found that DAA-associated HCV eradication improved splenomegaly. Thrombocytopenia in HCV-infected patients is thought to be due to portal hypertension-induced splenomegaly, resulting in the splenic pooling of platelets [23]. Spleen size and thrombocytopenia are thought to be associated, and meta-analyses have shown that splenectomy in addition to hepatectomy or liver transplantation improved thrombocytopenia and/or leukocytopenia compared with hepatectomy or liver transplantation alone [24, 25]. Reduced portal hypertension following trans-jugular intrahepatic portosystemic shunt was also found to improve thrombocytopenia [26, 27]. Liver transplantation has been reported to normalize platelet and leukocyte counts, reduce splenomegaly and improve portal hypertension [28, 29]. Collectively, HCV-induced liver inflammation and fibrosis contribute to portal hypertension and splenomegaly, with splenomegaly also contributing to thrombocytopenia and leukocytopenia. Therefore HCV eradication with DAA could improve this sequence of events.

DAA treatment was also shown to result in rapid and significant improvements in liver elasticity [30–32], suggesting that liver elasticity, as determined by liver elastography, was largely affected by liver inflammation. Liver elasticity is dependent on the elastography method, with acoustic radiation force impulse (ARFI) regarded as a better determinant of liver fibrosis than transient elastography [33]. DAA eradication of HCV has been shown to improve liver elasticity within 12 weeks, as determined by transient elastography. ARFI, however, showed improvements in liver elasticity, and maintenance of spleen elasticity, for 3 years after DAA [34]. Although few studies to date have evaluated changes in spleen size after DAA, a case study showed that spleen size was reduced 2 years after DAA eradication of HCV [35], a finding consistent with the present results. Collectively, these findings show that splenomegaly may be improved by clearance of hepatic inflammation within 2 years after DAA treatment, with further improvements resulting from a decrease in hepatic fibrosis.

The present study had several limitations. First, the number of patients included in the study was limited and varied, and the retrospective design may have led to selection bias and some errors in the measurement of SI. Liver fibrosis progression by liver biopsy or elastography could be assessed only in some of these patients, with cirrhosis diagnosed by individual physicians, not centrally. Second, splenomegaly in these patients was assessed by measuring SI with area. Spleen volume or spleen elasticity may better reflect hypersplenism, as these measures are more objective and functional than SI. Third, serum TPO concentration and HCV-RNA titers in bone marrow could not be evaluated in the present study, making it difficult to determine whether thrombocytopenia was due to myelosuppression or platelet destruction. Fourth, studies have shown that treatment of patients with advanced liver fibrosis and/or severe portal hypertension does not result in full recovery of liver function or complete abrogation of portal hypertension [36–38]. In the present study, liver fibrosis could not be quantified and portal hypertension could not be determined. Future detailed studies are required.

## Conclusions

DAA treatment of HCV infected patients resulted in the rapid clearance of hepatic inflammation and improvement in bone marrow suppression due to HCV infection. HCV eradication by DAA improved liver fibrosis and portal hypertension, resulting in a gradual reduction in spleen size.

## Supplementary Information

**Additional file1: Supplementary Table 1. **Results of treatment with DAAs. **Supplementary Table 2.** Improvement of esophageal varices after DAA treatment. **Supplementary Fig 1.** A flow chart of present study. HCV, hepatitis C virus, DAA direct acting antiviral, SI, splenic index. **Supplementary Fig 2.** A) Difference between each measurement of splenic index by ultrasonography. **Supplementary Fig 3.** Correlation between splenic indix measured by ultrasonographyand splenic index measured by CT. **Supplementary Fig 4.** A) Changes of platelets count after HCV eradication with DAA treatment according to baseline platelet count. **Supplementary Fig 5.** A) Changes of white blood cells count within 24 weeks after HCV eradication with DAA treatment. **Supplementary Fig 6.**  Changes of Fib-4 index within 24 weeks after HCV eradication with DAA treatment.

## Data Availability

The data that support the findings of this study are available upon reasonable request from the corresponding author.
