# Supplementary material for: Long term changes in thrombocytopenia and leucopenia after HCV eradication with direct-acting antivirals
Source: BMC Gastroenterol. 2023 May 25;23:182. doi: 10.1186/s12876-023-02829-w (PMC10210476; doi:10.1186/s12876-023-02829-w)
Supplement: Supplementary file 1 — Additional file1: Supplementary Table 1. Results of treatment with DAAs. Supplementary Table 2. Improvement of esophageal varices after DAA treatment. Supplementary Fig 1. A flow chart of present study. HCV, hepatitis C virus, DAA direct acting antiviral, SI, splenic index. Supplementary Fig 2. A) Difference between each measurement of splenic index by ultrasonography. Supplementary Fig 3. Correlation between splenic indix measured by ultrasonographyand splenic index measured by CT. Supplementary Fig 4. A) Changes of platelets count after HCV eradication with DAA treatment according to baseline platelet count. Supplementary Fig 5. A) Changes of white blood cells count within 24 weeks after HCV eradication with DAA treatment. Supplementary Fig 6. Changes of Fib-4 index within 24 weeks after HCV eradication with DAA treatment. [file 12876_2023_2829_MOESM1_ESM.pdf]

Supplementary Table 1. Results of treatment with DAAs

Regimen	Case with SVR/with treatment, (SVR percent)
DCV/ASV	33/35, (94)
OBV/PTV/r	2/2, (100)
SOF/rib	9/10, (90)
SOF/LDV	48/49, (98)
EBR/GZR	2/3, (67)
GLE/PIB	11/11, (100)
SOF/VEL	5/5, (100)

DAA, direct acting antiviral; SVR, sustained viral response; DCV, daclatasvir: ASV, asunaprevir; OBV, ombitasvir; PTV, paritaprevir; r, ritonavir; SOF, sofosbuvir; rib, ribavirin; LDV, ledipasvir; EBR, elbasvir; GZR, grazoprevir; GLE, glecaprevir; PIB, pibrentasvir; VEL, velpatasvir.

Supplementary Table 2. Improvement of esophageal varices after DAA treatment

Factors	Improvement of esophageal varices		p
	Present (n=5)	Absent (n=33)	
Age (yrs)	68.2±9.0	71.8±7.4	0.637
ALT (U/L)	50±33	61±55	0.614
γ-GTP (U/L)	51±37	44±37	0.854
Albumin (g/dL)	3.4±0.4	3.5±0.4	0.892
Total-bilirubin (mg/dL)	1.1±0.3	0.9±0.4	0.236
Creatinine (mg/dL)	0.8±0.2	1.2±1.7	0.315
White blood cells (/μL)	3722±1627	4033±1450	0.842
Platelets ($x10^4$/μL)	9.7±4.0	10.6±4.7	0.809
AFP (ng/mL)	9.9±5.2	15.2±16.8	0.219
Fib-4 index	7.16±3.58	7.02±3.81	0.814
Splenic Index (cm^2)	44.7±33.3	38.0±18.4	0.207
Splenic Index 4 years after DAA treatment	28.1±18.4	51.1±51.1	0.018

Statistical difference was compared with Mann-Whitney U-test. DAA, direct acting antiviral; yrs, years; ALT, alanine amino transferase; γ-GTP, γ-glutamyl transpeptidase; AFP, alpha fetoprotein

Supplementary Figure 1

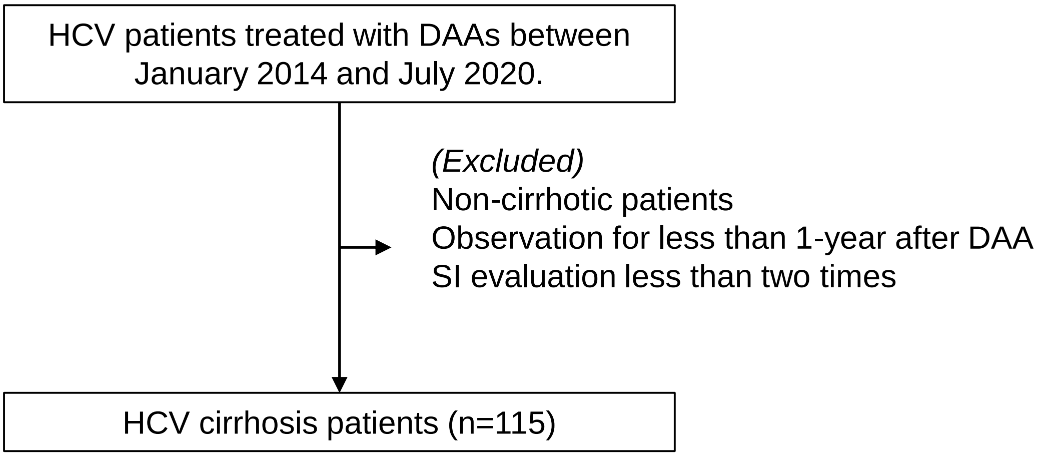

Supplementary Fig. 1. A flow chart of present study. HCV, hepatitis C virus; DAA, direct acting antiviral; SI, splenic index.

Supplementary Figure 2

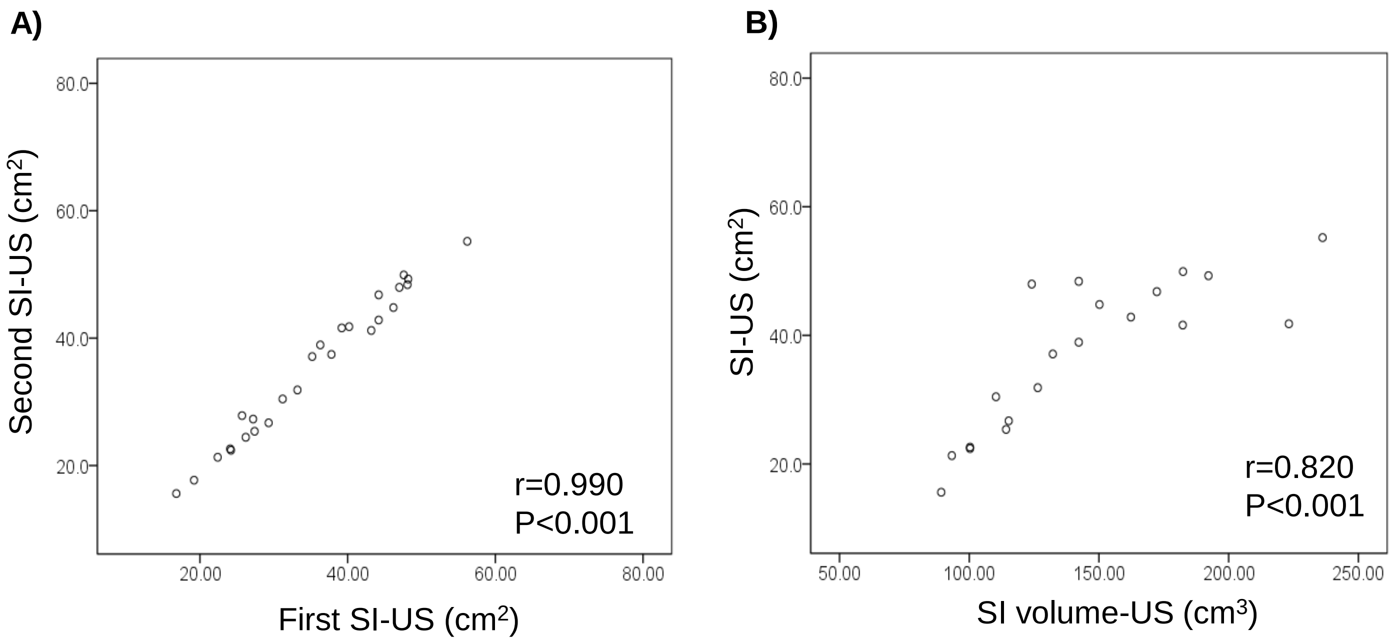

Supplementary Fig. 2. A) Difference between each measurement of splenic index by ultrasonography. The first measurement of splenic index was shown in X-axis (First SI-US), whereas the second measurement of splenic index was shown in Y-axis (Second SI-US).

B) Correlation between splenic index measured by ultrasonography (SI-US) and splenic index volume measured by US (SI volume-US).

Numbers at right lower column represent correlation coefficient (r) and statistical significance (P).

Supplementary Figure 3

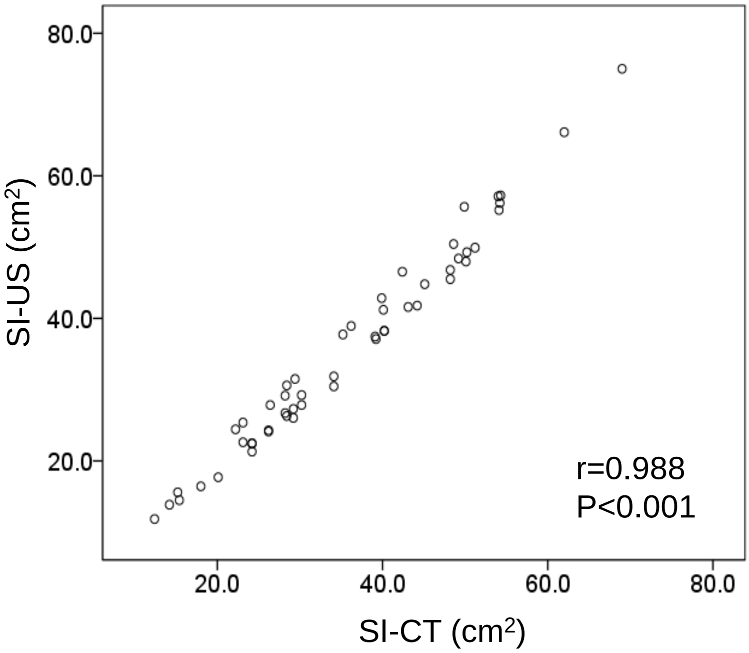

Supplementary Fig. 3. Correlation between splenic index measured by ultrasonography (SI-US) and splenic index measured by CT (SI-CT). Numbers at right lower column represent correlation coefficient (r) and statistical significance (P).

Supplementary Figure 4

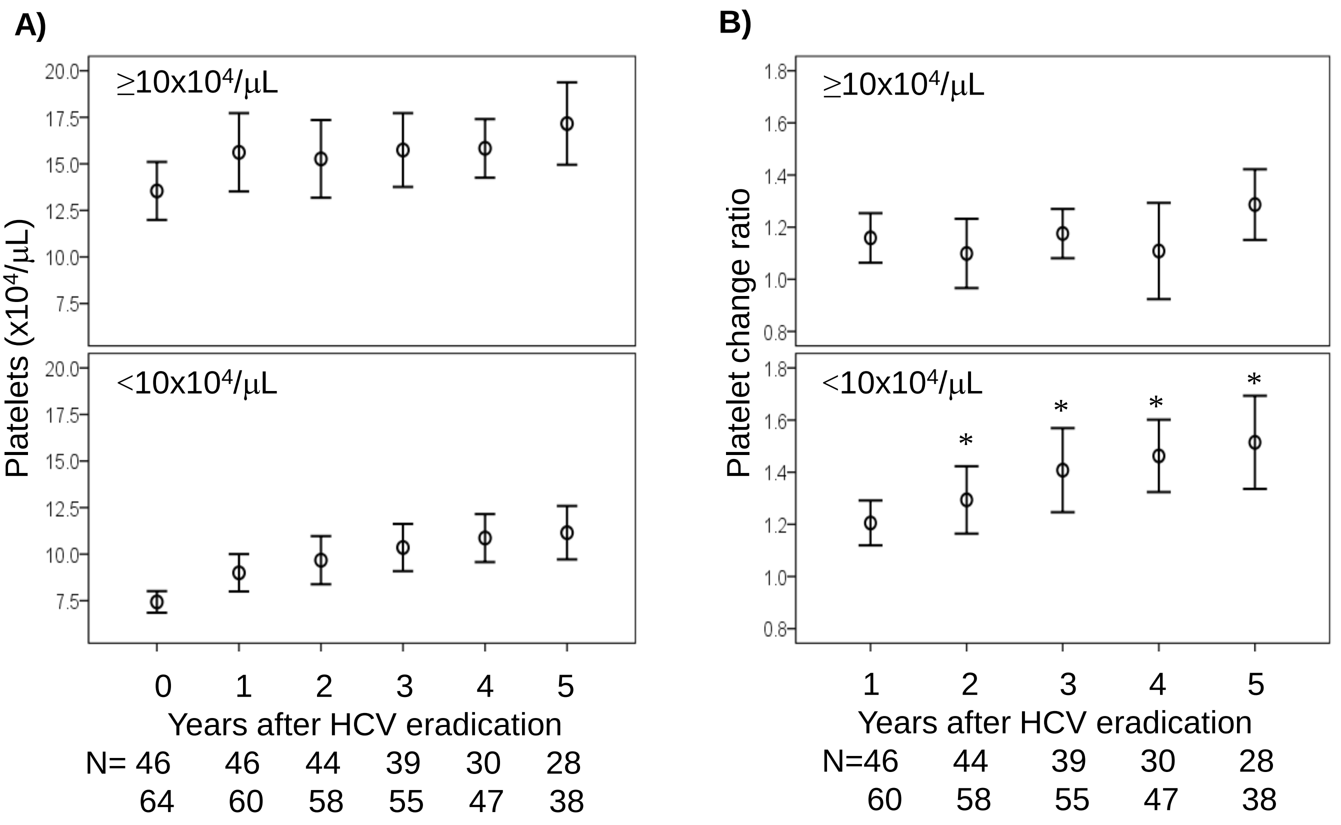

Supplementary Fig. 4. A) Changes of platelets count after HCV eradication with DAA treatment according to baseline platelet count. Upper graph represents those with patients ≥10x10⁴/μL, whereas lower graph represent those with <10x10⁴/μL. B) Changes of platelets change ratio with baseline after HCV eradication with DAA treatment according to baseline platelet count. Upper graph represents those with patients ≥10x10⁴/μL, whereas lower graph represent those with <10x10⁴/μL. Numbers under graphs mean the number of patients. Upper numbers represent the number of patients with ≥10x10⁴/μL, whereas lower represent those with <10x10⁴/μL.

.

Supplementary Figure 5

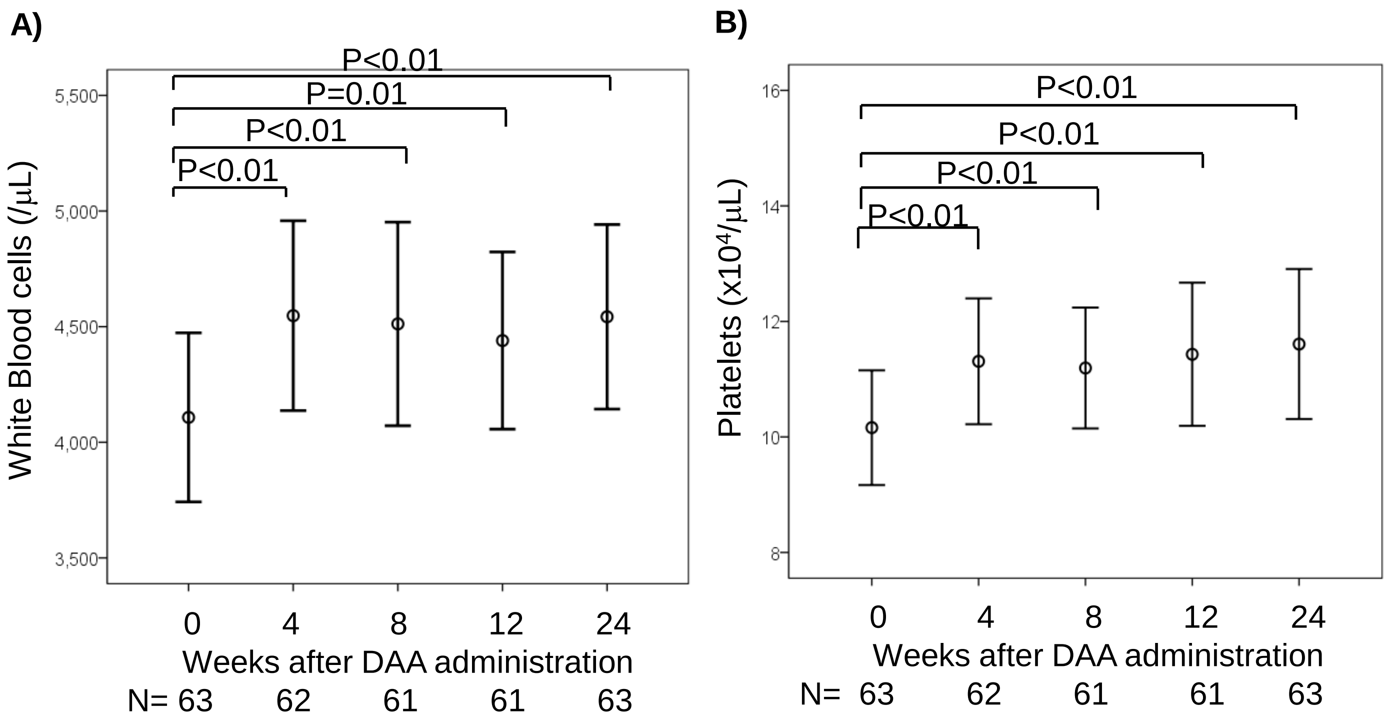

Supplementary Fig. 5. A) Changes of white blood cells count within 24 weeks after HCV eradication with DAA treatment. B) Changes of platelets count within 24 weeks after HCV eradication with DAA treatment. Numbers under graphs mean the number of patients. These rapid changes could be evaluated in 63 patients with HCV-cirrhosis who achieved SVR.

.

Supplementary Figure 6

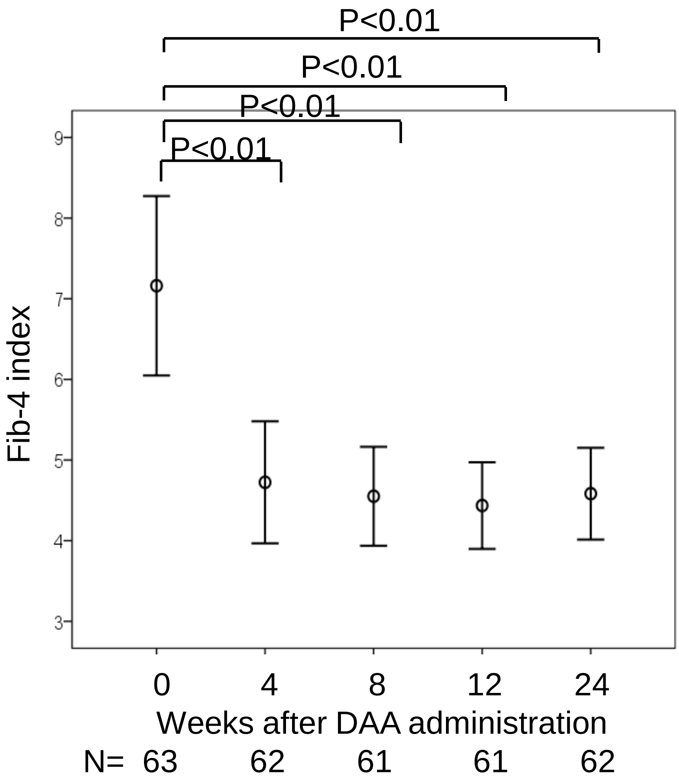

Supplementary Fig. 6. Changes of Fib-4 index within 24 weeks after HCV eradication with DAA treatment. Numbers under graphs mean the number of patients.

.
